# Human Exposure to Live Poultry and Psychological and Behavioral Responses to Influenza A(H7N9), China

**DOI:** 10.3201/eid2008.131821

**Published:** 2014-08

**Authors:** Liping Wang, Benjamin J. Cowling, Peng Wu, Jianxing Yu, Fu Li, Lingjia Zeng, Joseph T. Wu, Zhongjie Li, Gabriel M. Leung, Hongjie Yu

**Affiliations:** Division of Infectious Disease, Key Laboratory of Surveillance and Early-warning on Infectious Disease, Chinese Center for Disease Control and Prevention, Beijing, China (L. Wang, J. Yu, F. Li, L. Zeng, Z. Li, H. Yu);; Division of Epidemiology and Biostatistics, School of Public Health, Li Ka Shing Faculty of Medicine, The University of Hong Kong, Hong Kong Special Administrative Region, China (B.J. Cowling, P. Wu, J.T. Wu, G.M. Leung)

**Keywords:** influenza, viruses, influenza A(H7N9), live poultry, exposure, transmission, psychological, behavioral, survey, China, respiratory infections

## Abstract

To investigate human exposure to live poultry and changes in risk perception and behavior after the April 2013 influenza A(H7N9) outbreak in China, we surveyed 2,504 urban residents in 5 cities and 1,227 rural residents in 4 provinces and found that perceived risk for influenza A(H7N9) was low. The highest rate of exposure to live poultry was reported in Guangzhou, where 47% of those surveyed reported visiting a live poultry market >1 times in the previous year. Most (77%) urban respondents reported that they visited live markets less often after influenza A(H7N9) cases were first identified in China in March 2013, but only 30% supported permanent closure of the markets to control the epidemic. In rural areas, 48% of respondents reported that they raised backyard poultry. Exposure to live commercial and private poultry is common in urban and rural China and remains a potential risk factor for human infection with novel influenza viruses.

## Abstract 2

Exposure was common in urban and rural areas and remains a potential risk factor for human infection.

The novel influenza A(H7N9) virus was identified in early 2013; as of March 31, 2014, a total of 404 laboratory-confirmed cases of human infection had been reported. These cases included 394 in mainland China, 2 in Taiwan, 7 in Hong Kong, and 1 in Malaysia (*1*,*2*). Only 2 laboratory-confirmed cases were identified in the summer months (June–September 2013), but beginning in early October 2013, the virus reemerged and caused many new human infections (*3*,*4*).

Previously published studies have reported that most human infections appear to have occurred as a result of exposure to live poultry, particularly through visits to live poultry markets (LPMs) in urban areas (*3*,*5*–*8*). No published reports have detailed population exposure to live poultry and LPMs in influenza A(H7N9) virus–affected areas in China, and few data on live poultry exposure have been previously reported in areas in which the virus has not been detected (*4*,*9*,*10*). In addition, little information has been reported on how the population of China responded to the outbreak and the control measures that were implemented. To clarify responses to the influenza A(H7N9) outbreak in China, we investigated patterns in human exposure to live poultry in LPMs and at home, examined risk perception and behavioral responses in the population, and compared these parameters between urban and rural areas in China that were affected or unaffected by the virus.

## Methods

### Study Design

We collected information on human exposure to poultry, risk perception and psychological responses to the outbreak, preventive behaviors, and attitudes toward control measures, including closure of LPMs. We used 2 approaches to collect these data. In urban areas, we conducted telephone surveys because access to mobile telephones is high, making the approach feasible. In rural areas, where telephone accessibility is lower, we conducted door-to-door surveys.

**Figure 1:**
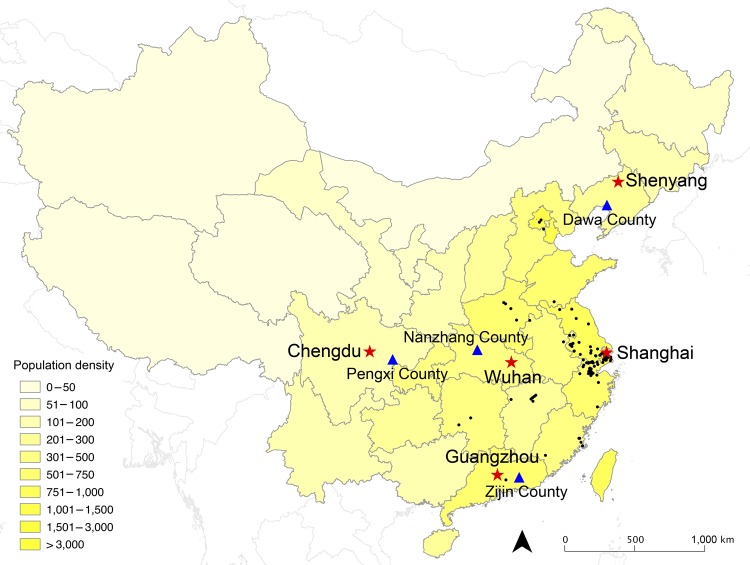
Geographic distribution of urban locations (red stars) and rural locations (blue triangles) selected for population survey to determine human exposure to live poultry and attitudes and behavior toward influenza A(H7N9) in China, 2013. Black dots indicate geographic locations of laboratory-confirmed cases of H7N9 through October 31, 2013. Shading indicates population density (persons per square kilometer). The 5 selected urban locations were Chengdu, capital of Sichuan Province in western China, population 10 million; Guangzhou, capital of Guangdong Province in southern China, population 13 million; Shanghai, a municipality in eastern China, population 23 million; Shenyang, capital of Liaoning Province in northeastern China, population 8 million; and Wuhan, capital of Hubei Province in central China, population 10 million. The 4 rural areas were Dawa County (Panjin city, Liaoning Province), Zijin County (Heyuan city, Guangdong Province), Nanzhang County (Xiangfan city, Hubei Province), and Pengxi County (Suining city, Sichuan Province).

We selected 5 large cities for our study to represent diverse levels of socioeconomic development and geographic location: Chengdu, Guangzhou, Shanghai, Shenyang, and Wuhan (Figure 1). Before our study, no laboratory-confirmed human cases of influenza A(H7N9) had been reported in these cities except for Shanghai; 1 environmental sample had tested positive for the virus in Guangzhou (*11*). In each city, we aimed to interview >500 adult residents (>18 years of age) who had been living there for >1 year. The telephone surveys were conducted by using a computer-assisted interviewing system, which enabled random generation of mobile telephone numbers and systematic data collection across each city. On each call, after the study was explained and verbal consent obtained, the respondent would be recruited into the study and asked to complete the survey. If a respondent were busy, a call would be made later, when the respondent was available to finish the questionnaire. Unanswered numbers were given 4 follow-up calls, made at different hours and on different days of the week, before being classified as invalid. The online Technical Appendix shows the survey used in English and Chinese.

Although we had planned to use the same telephone survey approach in rural areas, a pilot study revealed it was not feasible because the survey would occur during the busy farming season, when residents would not be readily available by telephone. Instead, in rural areas we conducted door-to-door surveys. In mainland China, some cities/counties that are administrated as rural regions actually include semiurban areas, such as towns in a county, and rural areas, such as villages in a town/county. The living conditions and lifestyle of residents in semiurban areas are similar to those of urban residents, whereas residents in rural areas live in a different environment, with low population density and a more self-sustainable life, mainly dependent on farming. We used convenience sampling to choose 4 counties from rural rather than semiurban areas. Rural sites were selected on the basis of the level of economic development (measured by gross domestic product per capita) and the overall incidence of infectious diseases in 2012. Given the tiers of administration levels in mainland China, including province, city, county, town, and village, we selected a city from each of the 4 provinces with mid-level gross domestic product per capita compared with other cities in the province and with an incidence of notifiable infectious diseases above the provincial average. Within each province, we then selected a rural county from each of the 4 cities areas. As a result, we chose Dawa County (Panjin city, Liaoning Province), Zijin County (Heyuan city, Guangdong Province), Nanzhang County (Xiangfan city, Hubei Province), and Pengxi County (Suining city, Sichuan Province) for the study (Figure 1). At time of the survey, none of these counties had laboratory-confirmed human infections with avian influenza A(H7N9) virus. 

After the initial selections, all towns within a county were stratified into high, middle, and low levels of socioeconomic status on the basis of census data (*12*–*15*), and 1 town was selected at random within each strata. Then, 2 villages were selected at random within each town, a convenience sample of 50 households was recruited in each village, and 1 adult in each household (>18 years of age and resident in the village for >1 year) was interviewed. To improve cooperation, each rural interviewee received a small gift worth ≈10 Chinese renminbi (6.1 renminbi = $1 US), such as a towel or a bottle of shampoo, after the survey was completed. All selected participants in the rural areas consented to be interviewed during the survey. The time taken to complete the survey was 16 minutes on average for each participant.

The urban surveys were conducted in May and June 2013 and the rural surveys in July and August 2013. Ethical approval was obtained from the Institutional Review Board of the Chinese Center for Disease Control and Prevention before the survey was conducted.

### Survey Instrument

All surveys in urban and rural areas were conducted by using the same questionnaire, which was based on an instrument used during the outbreaks of severe acute respiratory syndrome (SARS) in 2003 (*16*,*17*) and influenza A(H1N1)pdm09 in 2009 (*18*). The survey instrument was pretested for face and content validity, length, and comprehensibility. Most answers were ranked on ordinal Likert scales. We used the State Trait Anxiety Inventory to measure the general level of anxiety in the population (*16*–*18*). 

We investigated exposure to live poultry in backyards and in LPMs, which are defined as markets where the public can buy live chickens, ducks, pigeons, and other birds. Because LPMs are rare in rural areas and rural residents seldom visit LPMs, we did not ask rural respondents about exposures to live poultry in LPMs, only about backyard poultry exposure. In urban areas, we asked respondents about frequency of visits to LPMs and behaviors in LPMs (i.e., frequency of purchases, practice of picking up birds before purchasing, location where purchased live poultry were slaughtered). We asked all respondents about perception of risk for influenza A(H7N9) infection and perceived severity of such an infection, preventive practices in general and specifically in response to influenza A(H7N9), and attitudes toward influenza A(H7N9) and closure of LPMs. 

### Statistical Analysis

Statistical analyses were conducted in R version 2.13.0 (R Foundation for Statistical Computing, Vienna, Austria). We performed descriptive analyses of responses in each location and compared responses between urban areas with and without laboratory-confirmed cases of influenza A(H7N9) by using χ^2^ tests. For the subset of respondents who reported purchasing live poultry in LPMs during the previous year, we used a multivariate logistic regression model to estimate the associations of age, sex, educational level, and geographic location with attitudes toward closure of LPMs and changes in habits of buying live poultry after public health authorities announced the first human influenza A(H7N9) case on March 31, 2013 (*19*).The sample size of 500 respondents in each city and 300 respondents in each rural county was chosen to ensure precision of answers to within ±4% and ±6%, respectively, and to ensure reasonable statistical power to identify differences in responses of 5%–10% or more between locations.

## Results

**Figure 2:**
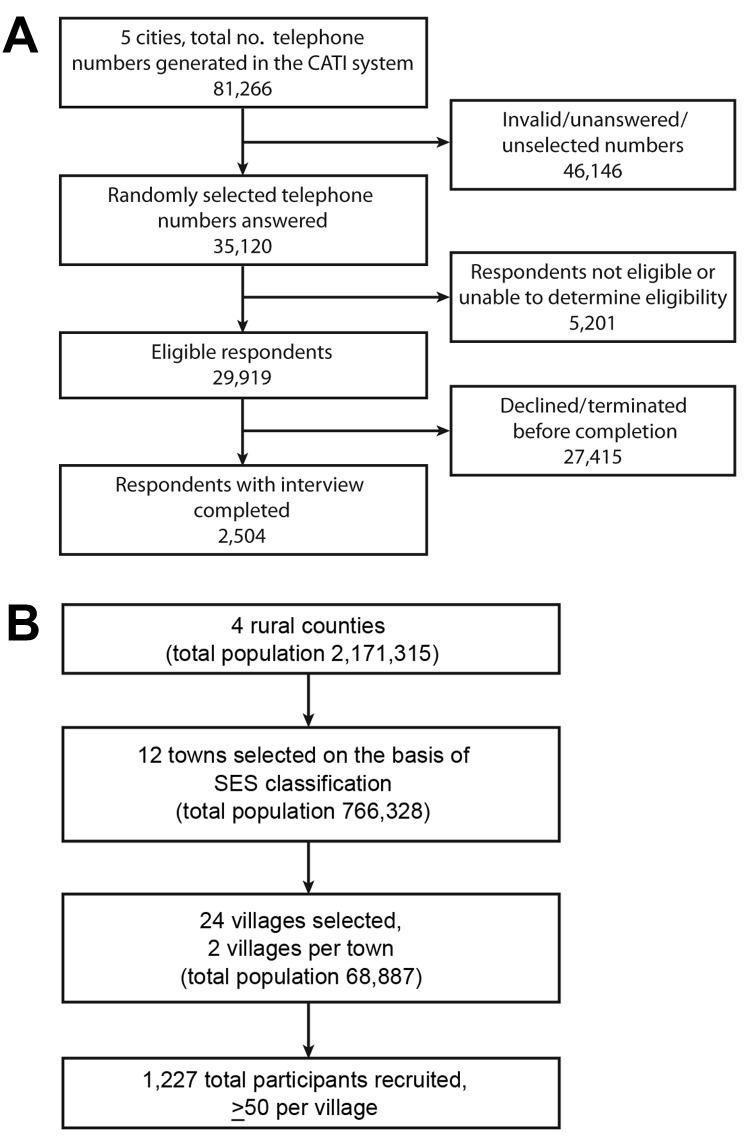
Flowcharts for recruitment of participants for telephone surveys and face-to-face interviews to determine human exposure to live poultry and attitudes and behavior toward influenza A(H7N9) in China, 2013. A) Flowchart for telephone surveys conducted in 5 urban areas: Chengdu (capital of Sichuan Province), Guangzhou (capital of Guangdong Province), Shanghai municipality, Shenyang (capital of Liaoning Province), and Wuhan (capital of Hubei Province). B) Flowchart for face-to-face interviews conducted in 3 rural areas: Dawa county (Panjin city, Liaoning Province), Zijin county (Heyuan city, Guangdong Province), Nanzhang county (Xiangfan city, Hubei Province), and Pengxi county (Suining city, Sichuan Province). CATI, computer-assisted telephone interview; SES, socioeconomic status.

In the 5 urban areas, 81,266 unique telephone numbers were dialed, and the overall response rate was 8% (number of participants [2,504] divided by number of calls with eligible respondents [29,919]) (Figure 2, panel A). The selection of 1,227 participants in 4 rural sites is illustrated in Figure 2, panel B. The surveys were conducted from May 23 through August 24. During this period, the influenza A(H7N9) epidemic had passed its peak, and few cases occurred. Guangdong Province notified its first human influenza A(H7N9) case on August 9, after the completion of the survey in Guangzhou on June 26.

Respondents in urban areas tended to have white-collar jobs or were unemployed, were younger, had more education and higher income, and were less likely to be married than those in rural areas (Table 1). However, because the surveys were conducted in different forms in urban versus rural areas and the general characteristics of participants were different, including the risk for becoming infected with influenza A(H7N9) virus and the types of potential exposure to avian influenza viruses, we did not make any further direct quantitative comparisons between urban and rural respondents. For comparisons among urban areas, respondents were generally similar, but reported incomes were higher for Shanghai and Guangzhou than for the other 3 cities (data not shown).

**Table 1:** Sociodemographic characteristics of participants recruited for urban and rural surveys of influenza A(H7N9) awareness, China, 2013*

Characteristic	No. (%) persons	
	Urban, n = 2,504	Rural, n = 1,227
Male sex	1,288 (51.4)	626 (51.0)
Age group, y		
18–44	1,938 (77.5)	685 (55.8)
45–64	415 (16.6)	405 (33.0)
>65	147 (5.9)	137 (11.2)
Educational attainment		
No formal education	38 (1.5)	86 (7.0)
Primary school	191 (7.6)	259 (21.1)
Middle school	391 (15.6)	464 (37.9)
High school	593 (23.7)	268 (21.9)
College and above	1,291 (51.6)	148 (12.1)
Occupation		
Service workers and shop sales workers	601 (24.0)	164 (13.4)
Professionals	504 (20.1)	66 (5.4)
Retired	293 (11.7)	61 (5.0)
Unemployed	678 (27.1)	195 (15.9)
Full-time students	232 (9.3)	111 (9.0)
Homemakers	96 (3.8)	86 (7.0)
Agricultural and fishery workers	100 (4.0)	544 (44.3)
Marital status		
Single	941 (38.1)	269 (22.0)
Married	1,458 (59.0)	923 (75.4)
Divorced/separated	35 (1.4)	12 (1.0)
Widowed	36 (1.5)	20 (1.6)
Average household income, in renminbi*		
No income	65 (3.0)	83 (6.8)
<3,000	368 (17.0)	748 (61.2)
3,001–6,000	627 (28.9)	264 (21.6)
6,001–10,000	408 (18.8)	80 (6.5)
10,001–50,000	396 (18.2)	28 (2.3)
Not sure	307 (14.1)	20 (1.6)
Recent history of travel away from home		
Yes	479 (19.1)	117 (9.6)

*6.1 Chinese renminbi = $1 US.

**Figure 3:**
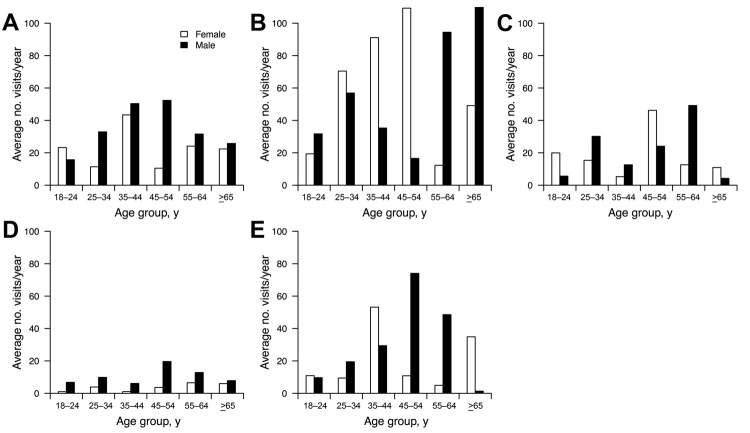
Age- and sex-specific patterns in exposures to live poultry markets in 5 urban areas of China, 2013. A) Chengdu; B) Guangzhou; C) Shanghai; D) Shenyang; E) Wuhan.

We assessed exposures to live poultry and visits to LPMs in the 5 cities. In total, 33% of respondents reported visiting LPMs during the preceding year, the highest proportion in Guangzhou; notable differences were found between cities (Table 2). By imputing midpoints of reported purchasing rates, we estimated that the mean number of live poultry purchased per year varied between cities: 6.8 for Shenyang, 19 for Shanghai, 20 for Wuhan, 28 for Chengdu, and 47 for Guangzhou. Age-specific patterns in exposure to live poultry were generally similar for men and women within each city, with some exceptions. In Guangzhou, women 35–54 years of age purchased poultry in LPMs much more frequently than did men of the same age, but the reverse was true for those >65 years of age (Figure 3). We found no evidence of a substantial difference in poultry exposures by sex in Shanghai (Figure 3). 

**Table 2:** Exposure to live poultry and attitudes toward closure of LPMs among participants recruited in urban areas for surveys related to influenza A(H7N9) awareness, by area, China, 2013*

Exposure	No. (%) persons					p value
	Chengdu, n = 500	Guangzhou, n = 500	Shanghai, n = 500	Shenyang, n = 504	Wuhan, n = 500	
Frequency of LPM visits in the previous year						<0.001
>1	183 (36.6)	237 (47.4)	161 (32.2)	97 (19.2)	151 (30.2)	
No. live poultry bought in the previous year†						<0.001
1–2/y	33 (18.0)	32 (13.5)	25 (15.5)	35 (36.1)	25 (16.6)	
3–5/y	31 (16.9)	27 (11.4)	30 (18.6)	23 (23.7)	28 (18.5)	
6–11/y	27 (14.8)	25 (10.5)	23 (14.3)	4 (4.1)	23 (15.2)	
1–3/mo	33 (18.0)	56 (23.6)	32 (19.9)	10 (10.3)	29 (19.2)	
1–2/wk	19 (10.4)	49 (20.7)	20 (12.4)	2 (2.1)	19 (12.6)	
3–5/wk	2 (1.1)	8 (3.4)	2 (1.2)	0	2 (1.3)	
Almost every day	2 (1.1)	4 (1.7)	2 (1.2)	0	2 (1.3)	
Almost none	36 (19.7)	36 (15.2)	27 (16.8)	23 (23.7)	23 (15.2)	
Pick up live poultry before buying‡						<0.001
Yes	120 (81.6)	136 (67.7)	94 (69.6)	38 (51.4)	97 (75.8)	
Where did you slaughter the live poultry?§						0.601
In LPM	123 (83.7)	175 (87.1)	119 (88.1)	66 (89.2)	113 (88.3)	
In household	22 (15.0)	23 (11.4)	15 (11.1)	6 (8.1)	13 (10.2)	
Other places	2 (1.4)	3 (1.5)	1 (0.7)	2 (2.7)	2 (1.6)	
Not buying or buying less since March 2013¶						<0.001
Yes	101 (68.7)	139 (69.2)	123 (91.1)	59 (79.7)	104 (81.3)	
Views toward closure of LPMs#						0.06
Agree	37 (25.2)	54 (26.9)	53 (39.3)	25 (33.8)	35 (27.3)	
Closure caused any inconvenience**						
More inconvenient	NA	NA	45 (31.5)	NA	NA	
Distance of nearest LPM from home, km						<0.001
<0.50	12 (13.3)	39 (31.0)	21 (18.9)	5 (13.5)	6 (15.0)	
0.51–1.00	23 (25.6)	42 (33.3)	32 (28.8)	4 (10.8)	10 (25.0)	
1.01–2.00	16 (17.8)	20 (15.9)	16 (14.4)	6 (16.2)	7 (17.5)	
>2.00	39 (43.3)	25 (19.8)	42 (37.8)	22 (59.5)	17 (42.5)	
Backyard poultry exposure	73 (14.6)	76 (15.2)	34 (6.8)	37 (7.3)	54 (10.8)	<0.001

*LPM, live poultry market; NA, not applicable. †Respondents who bought live poultry ≥1/year were further asked about the number of live poultry bought in the previous year, picking up poultry or not before buying, locations where poultry was slaughtered, and changes in poultry purchase behavior since influenza A(H7N9) outbreak.  ‡Respondents who answered always/usually to the question “Did you pick up poultry for examination before deciding to buy it?” were categorized as “Yes.”  §Respondents who stated that they always/usually have live poultry slaughtered in LPMs were categorized as “In LPM,” whereas those who answered always/usually in household were categorized as “in household.”  ¶Respondents who answered not buying since then/still buying but less than before to the question “Has your habit of buying live poultry changed since H7N9 was identified in China in March 2013?” were categorized as “Yes.”  #Respondents who answered strongly agree/agree to the question “Would you agree to permanent closure of live poultry markets in order to control avian influenza epidemics?” were categorized as “Agree.” **Respondents who reported that market closure caused great/some inconvenience were categorized as “More inconvenient.” This question was only asked of respondents in Shanghai because Shanghai was the only area where LPMs were closed at the time of the survey.

We further analyzed exposures in LPMs among urban residents on the basis of responses from the 829 (33%) of 2,504 participants who visited LPMs >1 time in the preceding year. Overall, 69% of these respondents reported that they always visited the nearest LPM; median distance from home to the nearest LPM was 1 km. Most respondents reported that they “usually” or “always” picked up poultry for examination before deciding to buy, with the highest proportion in Chengdu and lowest in Shenyang; 87% of respondents always arranged for slaughter of purchased poultry in the LPM, with no notable differences between cities.

During the study period, the general anxiety level among urban respondents (measured by the State Trait Anxiety Inventory) was low to moderate, but levels varied substantially between cities; the lowest mean scores were seen in Wuhan and Shenyang (Table 3). Perceived risk for influenza A(H7N9) in the following month (absolute susceptibility) and relative to others (relative susceptibility) were generally low in all cities, but highest in Shanghai. Respondents in Shanghai and Guangzhou were more likely to respond that they would be more worried than usual if they experienced an influenza-like illness (ILI). Twelve percent of respondents reported that they had worried about becoming ill with influenza A(H7N9) during the previous week; levels varied among cities, with a greater frequency of worry in Shanghai and Guangzhou (Table 3). Respondents in Shenyang reported the highest perceived severity of influenza A(H7N9) compared with seasonal influenza and avian influenza A(H5N1); respondents in Guangzhou reported the highest perceived severity of influenza A(H7N9) compared with that of SARS (Table 3).

**Table 3:** Risk perception related to influenza A(H7N9) among participants recruited for surveys in urban areas, by area, China, 2013*

Characteristic	Chengdu, n = 500	Guangzhou, n = 500	Shanghai, n = 500	Shenyang, n = 504	Wuhan, n = 500	p value†
Mean STAI scores (95% CI)	1.89 (1.85–1.94)	1.80 (1.75–1.84)	1.82 (1.78–1.86)	1.73 (1.69–1.77)	1.74 (1.71–1.78)	<0.001
Self-perceived susceptibility to influenza A(H7N9)‡						<0.001
High	13 (2.6)	9 (1.8)	14 (2.8)	1 (0.2)	5 (1.0)	
Even	61 (12.2)	98 (19.6)	61 (12.2)	54 (10.7)	90 (18.0)	
Low	426 (85.2)	393 (78.6)	425 (85.0)	449 (89.1)	405 (81.0)	
Perceived susceptibility to influenza A(H7N9) compared with others§						0.431
High	5 (1.0)	5 (1.0)	9 (1.8)	4 (0.8)	7 (1.4)	
Even	40 (8.0)	52 (10.4)	39 (7.8)	32 (6.3)	50 (10.0)	
Low	455 (91.0)	443 (88.6)	452 (90.4)	468 (92.9)	443 (88.6)	
ILI symptoms induced worry¶						<0.001
More	105 (21.0)	151 (30.2)	140 (28.0)	113 (22.4)	107 (21.4)	
Same as usual	197 (39.4)	198 (39.6)	192 (38.4)	165 (32.7)	233 (46.6)	
Less	198 (39.6)	151 (30.2)	168 (33.6)	226 (44.8)	160 (32.0)	
Infection with influenza A(H7N9) in next week#						0.004
Worry	64 (12.8)	68 (13.6)	68 (13.6)	49 (9.7)	53 (10.6)	
Think about it but no worry	77 (15.4)	57 (11.4)	104 (20.8)	92 (18.3)	78 (15.6)	
Never think about it	359 (71.8)	375 (75.0)	328 (65.6)	363 (72.0)	369 (73.8)	
Relative severity of influenza A(H7N9) compared with**						
Seasonal influenza	313 (62.6)	319 (63.8)	290 (58.0)	361 (71.6)	312 (62.4)	<0.001
Avian influenza A(H5N1)	159 (31.8)	163 (32.6)	170 (34.0)	203 (40.3)	156 (31.2)	0.028
SARS	52 (10.4)	57 (11.4)	54 (10.8)	45 (8.9)	51 (10.2)	0.779
Distance, km††	804	383	–	601	233	

*Values are no. (%) persons except as indicated. STAI, State Trait Anxiety Inventory; ILI, influenza-like illness; SARS, severe acute respiratory syndrome. †Differences between groups was examined with the Kruskal Wallis Test (assuming nonhomogeneous variances).  ‡Respondents who answered certain/very likely/likely to the question “How likely do you think it is that you will contract H7N9 avian flu over the next 1 month?” were categorized as “High”; those who answered never/very unlikely/unlikely were categorized as “Low.”  §Respondents who answered certain/much more /more to the question “What do you think is your chance of getting infected with H7N9 avian flu over the next 1 month compared to other people outside your family of a similar age?” were categorized as “High”; those who answered not at all/much less/less were categorized as “Low.”  ¶Respondents who answered extremely concerned/concerned much more than normal/concerned more than normal to the question “If you were to develop ILI symptoms tomorrow, would you be…?” were categorized as “More”; those who answered not at all concerned/much less concerned than normal/ concerned less than normal were categorized as “Less.”  #Respondents who answered worried about it all the time/worried a lot/worried a bit to the question “Did you worry about H7N9 in the past week?“ were categorized as “Worry.”  **Respondents who answered much higher/a little higher regarding the severity of influenza A(H7N9) compared with seasonal influenza, avian influenza A(H5N1), and SARS.  ††Distance between the survey location and the nearest area in which influenza A(H7N9) case(s) were reported.

In rural areas, as in urban areas, the mean State Trait Anxiety Inventory was low to moderate (Table 4). A total of 48% of respondents reported that they raised >1 type of poultry at home. Overall, 47% reported raising chickens, 15% raised ducks, and 8% raised geese; these proportions varied between counties (Table 4). In rural areas, levels of perceived absolute and relative susceptibility and concern about ILI or confirmed influenza A(H7N9) infection were generally low; some differences were seen between the 4 rural areas. Respondents in Nanzhang and Zijin were more likely to respond that they would be more worried than usual if they had an ILI; 24% of respondents in Zijin reported that they had worried about becoming ill with influenza A(H7N9) in the previous week, and the average level of worry in Zijin was higher than that for other counties (Table 4). Most respondents in each area perceived influenza A(H7N9) to be more severe than seasonal influenza but less severe than influenza A(H5N1) and SARS. 

**Table 4:** Risk perception related to influenza A(H7N9) and backyard poultry exposure among participants recruited for surveys in rural areas, by area, China, 2013*

Characteristic	Dawa, n = 310	Zijin, n = 308	Nanzhang, n = 308	Pengxi, n = 301	p value
Mean STAI scores (95% CI)	1.52 (1.47–1.57)	1.85 (1.80–1.90)	1.66 (1.62–1.70)	1.54 (1.48–1.61)	<0.001†
Self-perceived susceptibility to influenza A(H7N9)‡					<0.001
Higher	2 (0.6)	1 (0.3)	1 (0.3)	9 (3.0)	
Even	29 (9.4)	41 (13.3)	21 (6.8)	31 (10.3)	
Lower	279 (90.0)	266 (86.4)	286 (92.9)	261 (86.7)	
Perceived susceptibility to influenza A(H7N9) compared with others§					<0.001
Higher	0	1 (0.3)	2 (0.6)	8 (2.7)	
Even	10 (3.2)	25 (8.1)	3 (1.0)	36 (12.0)	
Lower	300 (96.8)	282 (91.6)	303 (98.4)	257 (85.4)	
Worry induced by ILI symptoms¶					<0.001
More	69 (22.3)	79 (25.6)	118 (38.4)	49 (16.3)	
Same as usual	73 (23.5)	113 (36.7)	118 (38.4)	113 (37.5)	
Less	168 (54.2)	116 (37.7)	71 (23.1)	139 (46.2)	
Infection with influenza A(H7N9) in next week#					<0.001
Worry	32 (10.3)	75 (24.4)	71 (23.1)	51 (16.9)	
Think about it but no worry	51 (16.5)	42 (13.7)	20 (6.5)	33 (11.0)	
Never think about it	227 (73.2)	190 (61.9)	217 (70.5)	217 (72.1)	
Severity of influenza A(H7N9) compared with**					
Seasonal influenza	201 (64.8)	181 (58.8)	224 (72.7)	182 (60.5)	0.001
Avian influenza A(H5N1)	105 (33.9)	112 (36.4)	67 (21.8)	92 (30.6)	<0.001
SARS	51 (16.5)	63 (20.5)	30 (9.7)	44 (14.6)	0.003
Distance, km††	482	2448	351	665	
Raising backyard poultry	141 (45.5)	135 (43.8)	166 (53.9)	168 (49.7)	0.067
Type of backyard poultry raised					
Chicken	120 (38.7)	134 (43.5)	162 (52.6)	161 (53.5)	<0.001
Ducks	49 (15.8)	45 (14.6)	20 (6.5)	65 (21.6)	<0.001
Geese	34 (11.0)	17 (5.5)	2 (0.6)	43 (14.3)	<0.001
Median no. live poultry raised	6	20	13	12	<0.001

*Values are no. (%) persons except as indicated. STAI, State Trait Anxiety Inventory; ILI, influenza-like illness; SARS, severe acute respiratory syndrome. †Differences between groups were examined with the Kruskal Wallis Test (assuming nonhomogeneous variances).  ‡Respondents who answered certain/very likely/likely to the question “How likely do you think it is that you will contract H7N9 avian flu over the next 1 month?” were categorized as “High”; those who answered never/very unlikely/unlikely were categorized as “Low.”  §Respondents who answered certain/much more /more to the question “What do you think is your chance of getting infected with H7N9 avian flu over the next 1 month compared to other people outside your family of a similar age?” were categorized as “High”; those who answered not at all/much less/less were categorized as “Low.”  ¶Respondents who answered extremely concerned/concerned much more than normal/concerned more than normal to the question “If you were to develop ILI symptoms tomorrow, would you be…?” were categorized as “More”; those who answered not at all concerned/much less concerned than normal/ concerned less than normal were categorized as “Less.”  #Respondents who answered worried about it all the time/worried a lot/worried a bit to the question “Did you worry about H7N9 in the past week?“ were categorized as “Worry.”  **Respondents who answered much higher/a little higher regarding the severity of influenza A(H7N9) compared with seasonal influenza, avian influenza A(H5N1), and SARS.  ††Distance between the survey location and the nearest area in which influenza A(H7N9) case(s) were reported.

Among respondents in urban areas who visited LPMs >1 time in the preceding year, 77% reported that they had stopped buying or bought lower amounts of live poultry since March 2013; this proportion was highest (91%) for Shanghai (Table 2). We examined factors affecting the likelihood of changing habits of buying live poultry and found greater changes among women, those with higher educational attainment, and those residing in Shanghai and Wuhan rather than in Chengdu. We found no statistically significant differences by age group (Table 5). 

**Table 5:** Factors associated with attitudes and behavior toward influenza A(H7N9) among survey respondents from urban areas who had visited a live poultry market during the previous year, China, 2013*

Characteristic	Odds ratio (95% CI)	
	Support closure of LPMs	Change purchase behavior
Sex		
F	1.19 (0.84–1.68)	**2.42 (1.61–3.63)**
M	Referent	Referent
Age group, y		
18–24	0.73 (0.37–1.45)	0.70 (0.36–1.36)
25–34	1.36 (0.85–2.17)	0.81 (0.49–1.34)
35–44	Referent	Referent
45–54	1.43 (0.72–2.83)	0.62 (0.3–1.26)
55–64	**3.28 (1.71–6.29)**	0.86 (0.39–1.9)
>65	**2.36 (1.04–5.32)**	1.42 (0.51–3.97)
Educational attainment		
Primary or below	Referent	Referent
Secondary	1.80 (0.92–3.50)	**1.95 (1.01–3.76)**
Tertiary or above	1.78 (0.90–3.53)	1.79 (0.91–3.51)
Urban sites		
Chengdu	Referent	Referent
Guangzhou	1.13 (0.69–1.85)	0.99 (0.62–1.60)
Shanghai	**1.77 (1.05–2.99)**	**4.89 (2.42–9.89)**
Shenyang	1.40 (0.74–2.64)	1.95 (0.97–3.95)
Wuhan	1.07 (0.62–1.86)	**2.05 (1.15–3.65)**

*Odds ratios were estimated by adjustment for all variables shown. Boldface indicates significance (p<0.05).

On average, across the 5 cities, 30% of respondents reported that they would support the closure of LPMs to control the epidemic; the proportion in support of closures was highest in Shanghai (39%) and lowest in Guangzhou (27%) and Chengdu (25%) (Table 2). We examined factors affecting the likelihood of supporting the closure of LPMs and found greater support among persons 55–64 years of age (odds ratio [OR] 3.28, 95% CI 1.71–6.29) and >65 years of age (OR 2.36, 95% CI 1.04–5.32). We also found greater support for closure of LPMs in Shanghai (OR 1.77, 95% CI 1.05–2.99) than in Chengdu but no significant differences by sex or educational attainment (Table 5). However, 32% of respondents in Shanghai reported that the closure of LPMs had caused them inconvenience.

## Discussion

We have reported empirical information on human exposures to live poultry, perception of risk for influenza A(H7N9), and behavioral responses to the 2013 influenza A(H7N9) outbreak in China. We found that exposure to LPMs in urban areas is common: 20%–50% of urban residents report >1 visit to an LPM in the preceding year (Table 2). Most respondents who purchased poultry in LPMs reported close contact with live poultry before slaughter. It is likely that the number of laboratory-confirmed cases of influenza A(H7N9) virus infection is lower than the actual number of human infections to date (*8*), and our results show that a broad cross-section of urban residents could be exposed to influenza A(H7N9) virus if it were prevalent among poultry in LPMs. In the spring 2013 outbreak, some evidence pointed to high prevalence of influenza A(H7N9) virus in certain LPMs (*6*), whereas official surveillance data from the Ministry of Agriculture identified the virus in only a small proportion of samples collected from across the country (of 4,488 samples tested, 0.9% were positive for the virus) (*11*). The absolute risk for human infection after close contact with poultry infected with the influenza A(H7N9) virus remains unclear.

We found that men in the 55–64-year age group had more exposures to live poultry than women in that age group, but no difference by sex among the small number of respondents ≥65 years of age in Shanghai (Figure 3). We had previously hypothesized that exposure to poultry in LPMs might be higher for older men than for older women (*3*). Our findings suggest that the higher risk for laboratory-confirmed influenza A(H7N9) virus infection among men during the spring 2013 outbreak in the Yangtze River Delta might not be explained by sex differences in exposure but rather by increased susceptibility to serious disease after infection among men (e.g., because of greater prevalence of co-existing conditions) or by increased access to health care and laboratory testing for men. However, our sample size was relatively small, particularly for respondents >65 years of age. As in a previous report of live poultry exposures in the southern China cities of Guangzhou in 2006 and Shenzhen in 2007 (*9*), we did not identify major differences in exposures among middle-aged adults compared with exposures among the elderly. However, most laboratory-confirmed influenza A(H7N9) cases have been in persons >60 years of age (*3*), consistent with our hypothesis that exposures in middle-aged adults may have led to milder disease that was less likely to result in laboratory testing (*3*,*9*).

A minority of respondents reported willingness to accept LPM closures in the event of future outbreaks of influenza A(H7N9). During the winter 2013–14 influenza season, in some areas where human cases of influenza A(H7N9) had been reported, local governments implemented short-term LPM closures; other administrations, including that of Shanghai, closed LPMs for longer periods. However, such interventions can have serious economic consequences. Given the lack of public support for LPM closure and the related economic concerns, whether to make additional closures should be considered carefully. Regular rest days (i.e., days on which live poultry are not sold and stalls must be disinfected and left empty of live birds) and bans on overnight retention of live poultry in markets have been successful in controlling the transmission of avian influenza viruses in LPMs in Hong Kong (*20*,*21*) and have been proposed in some areas of China (*8*).

Although almost all cases of influenza A(H7N9) cases have been identified in areas within or surrounding large cities, about half of the laboratory-confirmed avian influenza A(H5N1) cases in China were identified in rural residents, which indicates that avian influenza viruses can reach backyard poultry flocks and pose a risk to human health (*3*). Influenza A(H7N9) virus does not appear to have spread to backyard flocks at this time, however. Most confirmed human cases have occurred in urban areas among persons who have reported recent exposure to live poultry in LPMs, although a smaller number of cases occurred in persons who have reported recent exposure to backyard poultry (*3*). However, if the circulation of influenza A(H7N9) virus in backyard poultry were to increase, the number of potential exposures could be substantial because almost half of rural residents report raising backyard poultry. The risk for influenza A(H5N1) virus infection among rural residents has been reduced through better education about the danger of close contact with, or consumption of, sick or dead backyard poultry (*22*,*23*). Unfortunately, this approach would not be effective for controlling spread of influenza A(H7N9) virus because infected chickens do not show signs of illness.

Perception of risk for influenza A(H7N9) infection by respondents to our surveys was generally low, as might be expected given the small number of laboratory-confirmed cases in China. However, low perception of risk could pose difficulties for policy measures such as closure of LPMs. Indeed, we found generally low levels of public support for long-term closure of LPMs (Table 2), particularly in cities that had not been affected by influenza A(H7N9). Respondents in Guangzhou and Shanghai reported higher likelihood than residents of other cities that they would be worried if they showed signs and symptoms of ILI. This finding is unsurprising for Shanghai, but there had been no confirmed influenza A(H7N9) cases in Guangzhou at the time of our survey.

Our study has several limitations. First, the cross-sectional study design did not enable us to identify changes over time in risk perception or preventive behaviors. Having access to data on live poultry exposures before the identification of influenza A(H7N9) virus infections would have been helpful because the epidemic may have led to changes in exposure patterns by the time our survey was conducted. Second, because the survey was conducted by telephone in urban areas and face-to-face in rural areas, our results may have been affected by selection bias. We did attempt multiple calls to unanswered telephone numbers in an attempt to mitigate this bias, but the overall response rate for the telephone survey was low. Also, because the respondents self-reported their behaviors, the results might be affected by response biases (e.g., if respondents had incomplete recollection of past visits to LPMs). In particular, results could have been affected by social desirability bias if respondents felt uncomfortable reporting true patterns of poultry exposure or attitudes toward government interventions and preferred to report what they perceived to be ideal or most acceptable. 

Third, our analyses did not explore in depth the social or psychological factors underlying behavioral responses to influenza A(H7N9), such as the effect of perceived risk or severity. This area might be productive for further investigation. Fourth, similar to other cross-sectional knowledge–attitude–behavior studies, our survey could only provide descriptive data on live poultry exposure, risk perception, and behavioral changes. Inferences on the associations between different psychobehavioral factors will require further study. Furthermore, we did not investigate seasonal variation in poultry-purchasing behaviors, which could also be studied in longitudinal surveys.

In conclusion, exposures to live poultry are common in many areas of China. If influenza A(H7N9) virus were to become more prevalent among poultry, the number of human exposures could be substantial in the absence of control measures. Our findings highlight possible problems in the structure of the live poultry trade in China and the potential for improved protection of human and animal health (*8*,*24*).

Technical Appendix English and Chinese language versions of the questionnaire used for the telephone survey conducted in 5 cities in China to determine human exposure to live poultry and attitudes and behavior toward influenza A(H7N9), 2013.
