# Supplementary material for: Human Exposure to Live Poultry and Psychological and Behavioral Responses to Influenza A(H7N9), China
Source: Emerg Infect Dis. 2014 Aug;20(8):1296–305. doi: 10.3201/eid2008.131821 (PMC4111172; doi:10.3201/eid2008.131821)
Supplement: Technical Appendix — English and Chinese language versions of the questionnaire used for the telephone survey conducted in 5 cities in China to determine human exposure to live poultry and attitudes and behavior toward influenza A(H7N9), 2013. [file 13-1821-Techapp-s1.pdf]

Article DOI: http://dx.doi.org/10.3201/eid2008.131821

Human exposure to Live Poultry and Psychological and Behavioral Responses to Influenza A(H7N9), China

Technical Appendix

The following pages contain English and Chinese language versions of the questionnaire used for the telephone survey conducted in 5 cities in China to determine human exposure to poultry, risk perception, and psychological responses to influenza A(H7N9) outbreaks, preventive behaviors, and attitudes toward control measures, including closure of live poultry measures. At least 500 adult residents (age ≥18y) who had been living in the city for at least 1 year were interviewed. The telephone surveys were conducted using a Computer-Assisted Telephone Interviewing system, which enabled random generation of mobile telephone numbers and systematic data collection across each city. After explaining the study and obtaining verbal consent from respondents, they were recruited as study subjects and asked to complete the survey. If the respondents were busy at the time, a telephone call would be made later when the respondents were available to finish the questionnaire. Unanswered numbers were given 4 more follow-up calls, made at different hours and days of the week, before being classified as invalid.

H7N9 Avian Flu Survey in China

Version 1.0

Greetings: Hello, This is a national Tele-survey, conducted by Chinese Center for Disease Control and Prevention. We are collecting H7N9 Avian Flu-related information, which will be very important for the H7N9 control in China. It will take you around 10 minutes. Thank you for your cooperation, which will be highly appreciated.

SG1. Where do you live? *(Don't ask, could be recorded automatically)

① Beijing	② Shanghai
③ Shenyang	④ Wuhan
⑤ Guangzhou	⑥ Chengdu
⑦ Da'wa County, Panjin City	⑧ Nanzhang County, Xiangyang City
⑨ Pengxi County, Suining City	⑩ Zijin County, Heyuan City

SG2. How many years do you live here: _______(excluded, if reside less than 1 year.)

SG3. Gender *(Don't ask if obvious, must record)

① Female ② Male

SG4. What is your age?

a) *(required) ______ years old

b) ① 18-24 ② 25-34

③ 35-44 ④ 45-54

⑤ 55-64 ⑥ 65 or above

⑦ Refused

SG5. What is your occupation? *(required)

① Retired	② No job, unemployed, seeking job
③ Full-time students	④ Home makers
⑤ Professionals	⑥ Service workers and shop sales workers
⑦ Health care worker	⑧ Skilled agricultural and fishery workers; and occupations not classified
⑨ Farmer	⑩ Businessman selling live poultry or meat
⑪ Others	

Part 1 Self-Rated Health

PH1. How do you perceive your health in the past 1 week?

① Excellent ② Very good

③ Good ④ Fair

⑤ Poor

中国城市和农村人感染 H7N9 禽流感调查问卷[1]

2013 年 5 月 24 日 第 1.14 稿

开头语：您好，这里是中国疾控中心正在实施的全国性电话调查，以下问题将占用您约 10 分钟的时间，您的个人意见对于我国 H7N9 疫情的防控非常重要，谢谢您的配合！

（备留）。开头语：喂，先生/女士您好，我姓***，我是北京益派市场咨询有限公司的访问员，我们受国家疾控中心的委托进行一项全国性的调查，想了解公众对于 H7N9 禽流感的认知，我只会占用您几分钟时间，请您放心。您的电话号码是由我们通过电脑随机抽中的，您提供的资料将绝对保密，并只会用作综合分析。为保障数据的准确性，我们的访问将会被录音，但只用作内部参考，并会在短期内销毁（访问员注意：如被访者对这次访问有任何疑问或查询，访问员可说："如果您对今天的访问有任何疑问或查询，您可以拨打热线电话：****，或 010-58900546，与有关专家联系"）。

请问现在我们可以开始访问吗？可以，即开始；不可以，则结束。

SG1. 请问您是居住在 XX 吗？*(不必提问，CATI 自动记录)

① 北京	② 上海
③ 沈阳	④ 武汉
⑤ 广州	⑥ 成都
⑦ 辽宁盘锦市大洼县	⑧ 湖北襄阳市南漳县
⑨ 四川遂宁市蓬溪县	⑩ 广东河源市紫金县

SG2. 请问您在本地居住______年（少于 1 年则不满足调查条件）

SG2a. 您目前的居住状态：

① 与家人同住 ② 不与家人同住

SG3. 性别 *(如果已经很明显，不需再问，但请记录下来)

① 男性 ② 女性

[1]请自动生成调查时间及访问员姓名，并纳入数据库

SM1. Have you had any of the following signs or symptoms in the past 2 weeks?

		Yes	No	Don't know
a.	Fever ≥37.8℃(100℉) for 1 day or more	①	②	③
b.	Cough	①	②	③

SM2. If you had fever in the last 2 weeks (SM1a=yes), when did the fever start?

① __________ (dd-mm-yy)　　② Don't know

AX1. A number of statements which people have used to describe themselves are given below; please tell how you feel to the statements right now:

		Not at all	Sometimes	Moderately So	Very Much So
a.	I feel rested	①	②	③	④
b.	I feel content	①	②	③	④
c.	I feel comfortable	①	②	③	④
d.	I am relaxed	①	②	③	④
e.	I feel pleasant	①	②	③	④
f.	I feel anxious	①	②	③	④
g.	I feel nervous	①	②	③	④
h.	I am jittery	①	②	③	④
i.	I feel "high strung"	①	②	③	④
j.	I feel over-excited and "rattled"	①	②	③	④

BF4b. Please rate the current level of your worry towards H7N9 avian flu, 1 being very mild to 10 being very severe (1 = Very Mild, 10 = Very Severe) : ______.

Part 2 Exposure to live poultry markets (<u>Only for the urban cities</u>; go to EM7 directly for Beijing respondents)

EM1. How often did you go to live poultry markets in last year?

(Live poultry markets mean the markets where public could buy the live chicken, ducks and pigeons, etc)

① 1-2/year
② 3-5/year
③ 6-11/year
④ 1-3/month
⑤ 1-2/week
⑥ 3-5/week
⑦ Almost every day

SG4. 您的年龄?

a) *(必填) ______岁

b) ① 18-24　② 25-34
③ 35-44　④ 45-54
⑤ 55-64　⑥ 65 以上
⑦拒绝回答

SG5. 您的职业 *(必填)

① 退休　② 没有工作、失业、找工作中
③ 全日制学生　④ 家庭主妇
⑤ 专业人士　⑥ 服务行业或商店工作人员
⑦ 医务人员　⑧ 农业和渔业技工（不含禽类饲养）
⑨ 农民　⑩ 活禽饲养、贩卖人员或肉类销售人员
⑪ 其他

第一部分　健康自我评估

PH1. 请问过去 1 周您感觉自己身体状况如何？

① 非常好　② 很好
③ 好　④ 一般
⑤ 差

SM1. 过去 2 周，您是否出现以下症状？

		有	没有	不记得了
a.	持续 1 天或以上，摄氏 37.8℃以上的高烧	①	②	③
b.	咳嗽	①	②	③

SM2. 过去 2 周，如果您曾发过烧 (问题 SM1a 回答：有), 那么，您是从哪天开始发烧的?

① _____月_____日　② 不记得了

AX1. 下面是 10 个反映自我感觉的方面，请您分别回答最能表达您目前感觉的答案：

		完全没有	有些	中等程度	非常明显
a.	我感到安宁	①	②	③	④
b.	我感到满意	①	②	③	④
c.	我感到舒适	①	②	③	④
d.	我是放松的	①	②	③	④
e.	我感到愉快	①	②	③	④
f.	我感到焦虑	①	②	③	④
g.	我感到紧张不安	①	②	③	④
h.	我现在神经过敏	①	②	③	④
i.	我感到十分敏感和容	①	②	③	④

⑧ Almost not **(go to part 3)**

EM1a. The latest time you went to a wet market was __________ (mm-dd), or __________ days ago.

EM1b. How far is the wet market away from your residence? __________ kilometers

EM1c. Did you always used to go to the nearest wet market?

① Yes

② No, If no, how far is the wet market you went? __________ kilometers.

EM2. How many poultry did you buy in LPMs averagely in the whole year?

① 1-2/year

② 3-5/year

③ 6-11/year

④ 1-3/month

⑤ 1-2/week

⑥ 3-5/week

⑦ Almost every day

⑧ Almost not **(go to part 3)**

EM3. Are you accustomed to pick up the poultry for examination before deciding to buy it?

① Yes

② No

③ Sometime "yes", sometime "no"

EM4. Where was the live poultry slaughtered when you bought it?

① Always in wet market

② Usually in wet market

③ Usually in my household

④ Always in my household

⑤ Other places, _________

EM5. Whether your habit of buying the live poultry was changed since the first human H7N9 case was released in Mar 2013?

① Yes, not buying since then

② No, still buying and eating live poultry

③ Still buying but less than before

EM6. How do you think about the closure of wet market in order to control the H7N9 epidemic?

① Strongly agree

② Agree

③ Not Agree

	易激动				
j.	我感到过于激动和慌张	①	②	③	④

BF4b. 如果可以用 1—10 分来表达您对 H7N9 禽流感的担心程度，1 表示很少担心，10 表示非常担心，您目前担心程度是______分.

第二部分活禽市场暴露情况 （城市和农村调查内容不同）

（以下内容针对沈阳、北京、武汉、成都、上海和广州城市居民，其中北京居民直接跳 EM7 题）

EM1. 在过去一年中，您多久去一次活禽市场？

(活禽市场：主要是指公众去购买活禽活鸭等的市场)

① 每年 1-2 次

② 每年 3-5 次

③ 每年 6-11 次

④ 每月 1-3 次

⑤ 每周 1-2 次

⑥ 每周 3-5 次

⑦ 几乎每天

⑧ 不去（如果选择⑧，直接跳至第三部分）

EM1a. 您最近一次去活禽市场的时间是：___月___日 （或者 ___天以前）

EM1b. 您最近一次去的活禽市场距离您家有多远？________公里

EM1c. 您总是去距离最近的活禽市场吗？

① 是 ② 不一定，您常去的活禽市场距离您家多远？________公里

EM2. 一年里平均在活禽市场购买多少活禽？

① 每年 1-2 只

② 每年 3-5 只

③ 每年 6-11 只

④ 每月 1-3 只

⑤ 每周 1-2 只

⑥ 每周 3-5 只

⑦ 基本每天 1 只

⑧ 没买过（如果选择⑧，直接跳至第三部分）

EM3.在决定购买活禽前，您会自己挑选接触活禽吗？

①总是

②有时

③从不

EM4. 当您购买活禽后，会在哪里进行宰杀？

① 一直在活禽市场

② 通常在活禽市场

④ Strongly disagree
⑤ Don't know

EM7. Has the closure of LPMs caused you any inconvenience in your life? (Only ask the respondents from Beijing/Shanghai that has closed the markets)

① Great inconvenience ② Some inconvenience
③ Inconvenience a bit ④ No inconvenience
⑤ No inconvenience at all

Raising the backyard poultry at home (Only for the 4 rural cites)

EM8. Do you raise backyard poultry in the past year?
① Yes
② No (go to Part 3)

EM8.1. What type of backyard poultry do you raise at home? **(Multiple choice questions)**
① Chicken
② Ducks
③ Geese
④ Others______________.

EM8.2 How many backyard poultry do you have? (Including chicken, ducks, geese and others in total)?________

Part 3 Health Services Utilization

HS1 Have you used any of the following health services in the past 2 weeks?

		Yes		No
a.	Hospital (including 24-hour clinics/A&E services)	☐	______ times	☐
b.	Self- treatment (Drug/acupuncture)	☐	______ times	☐

Part 4 Contacts with Flu Cases

The following questions refer to the **past 2 weeks**:

CT1. Has anyone in your household had any flu symptoms in the past 2 weeks (e.g. fever, runny nose, cough, sore throat)?
① Yes, me (may include others) (please specify the ages of the members (oldest first): __ __ __ __ __)
② Yes, others but not me (please specify the ages of the members (oldest first): __ __ __ __ __)
③ No (go to Part 5 perception on H7N9 avian flu)

CT2. If yes, as a result of this did you personally take any preventive measures?

	Yes	No	Don't know
a. Wash hands more frequently	①	②	③

③ 通常在家中宰杀
④ 一直在家中宰杀
⑤ 其它地方

EM5. 2013 年 3 月底国家公布首例人感染 H7N9 禽流感病例以来，您家购买活禽的生活习惯是否有变？
①是，不购买了
②是，但还会买，只是比以前买的少一些
③否，仍然和以往一样的购买习惯

EM6. 为了控制 H7N9 禽流感疫情而永久关闭活禽市场，您如何看？
① 坚决支持
② 支持
③ 不支持
④ 坚决反对
⑤ 无所谓

EM7. 关闭活禽市场对您的日常生活带来不便吗？（仅询问北京、上海的市民）
① 极不方便 ② 有些不便
③ 稍微有点不便 ④ 几乎没有不方便
⑤ 完全没有不方便

（以下内容仅针对 4 个县的农村居民）

EM8. 在过去一年中，您家饲养鸡/鸭/鹅吗？ ①是 ②否（直接跳至第三部分）

EM8.1 您家饲养的家禽是？ ①鸡 ②鸭 ③鹅 ④其他_________

EM8.2 总共饲养多少只（含鸡/鸭/鹅/其它）？ ________________

下面将问您医疗服务使用的问题。

第三部分 医疗服务利用

HS1. 过去 2 周，您是否看过病或进行自我医疗？

		有		没有
1.	到医院门、急诊看病治疗	☐	______次数	☐
2.	到私人小诊所看病	☐	______次数	☐
3.	自我医疗，如服药，理疗等	☐	______次数	☐

b. Wear face mask	①	②	③
c. Isolation	①	②	③

CT3. As a result of this did the sick family member take any preventive measures?

	Yes	No	Don't know
a. Wash hands more frequently	①	②	③
b. Wear face mask	①	②	③
c. Isolation	①	②	③

CT4. How many days, after the onset of the first flu symptom, did the sick family member (include you if you are sick) seek medical advice?

Sick family members (oldest first)	Self	1	2	3	4	Additional
Days						
Did not seek medical advice						
Don't know						

Part 5 Perception on H7N9 Flu

BF1. How likely do you think it is that you will contract H7N9 avian flu over the next 1 month?

① Never ② Very unlikely
③ Unlikely ④ Evens
⑤ Likely ⑥ Very likely
⑦ Certain

BF2a. What do you think are your chances of getting H7N9 avian flu over the next 1 month compared to other people outside your family of a similar age?

① Not at all ② Much less
③ Less ④ Evens
⑤ More ⑥ Much more
⑦ Certain

BF3. Do you think H7N9 avian flu is spread by:

	Yes	No	Don't Know
a. Droplets	①	②	③
b. Air	①	②	③
c. Direct hand contact（e.g. via handshake）	①	②	③
d. Indirect hand contact (e.g. via doorhandle)	①	②	③
e. Oral-faecal	①	②	③
i. Cold weather	①	②	③
k. The air over long distances	①	②	③

第四部分 流感接触史（针对过去 2 周情况回答）

CT1. 过去 2 周中，您家中有没有人出现过流感症状（例如：发烧、咳嗽、流涕、咽痛）？（仅问与家人同住者）

①有，是我（可包括其他家庭成员）（跳至 CT2），
请填写出现流感症状者的年龄（年龄由大到小顺序：__________________________________）
②有，是其他家庭成员（跳至 CT2）
请填写出现流感症状者的年龄（年龄由大到小顺序：__________________________________）
③没有(直接跳至第五部分：对 H7N9 禽流感的认知)

CT1a. 过去 2 周中，您有没有出现过流感症状（例如：发烧、咳嗽、流涕、咽痛）？（仅问不与家人同住者）

①有（跳至 CT2，仅问 CT2）
③没有(直接跳至第五部分：对 H7N9 禽流感的认知)

CT2. 您是否采取了以下个人防护措施？

	是	否	不记得
a. 频繁洗手	①	②	③
b. 戴口罩	①	②	③
c. 自我隔离	①	②	③
d. 自行服用中药	①	②	③

CT3. 出现流感症状的家人是否采取了以下个人防护措施？

	是	否	不记得
a. 频繁洗手	①	②	③
b. 戴口罩	①	②	③
c. 隔离	①	②	③
d. 自行服用中药	①	②	③

CT4. 自出现流感症状当天算起，您或您的家人是在多长时间后去就诊的？(按照年龄顺序排列)

患病家庭成员（年龄由大到小排列）	1	2	3	4	5	其他成员
没去就诊						
发病到就诊的间隔（天）						
不记得了						

第五部分对 H7N9 禽流感认知

BF1. 您认为在接下来的 1 个月中，自己患上 H7N9 禽流感的可能性多大？

① 根本不可能 ② 很不可能
③ 不可能 ④ 说不好
⑤ 可能 ⑥ 很可能

	(e.g. from one building to another one)			
l.	Body contact with patients	①	②	③
m.	Touching objects that have been contaminated by the virus	①	②	③
n.	Close contact with chickens in a wet market	①	②	③

BF4. If you were to develop flu-like symptoms tomorrow, would you be

① Not at all worried ② Much less worried than normal
③ Worried less than normal ④ About same
⑤ Worried more than normal ⑥ Worried much more than normal
⑦ Extremely worried

BF4a. In the past one week, have you ever worried about catching H7N9 avian flu?

① No, never think about it ② Think about it but it doesn't worry me
③ Worries me a bit ④ Worries me a lot
⑤ Worry about it all the time

BF5a. How does H7N9 avian flu compare with seasonal flu in terms of seriousness?

① Much higher ② A little higher
③ Same ④ A little lower
⑤ Much lower

BF5d. How does H7N9 avian flu compare with H5N1 avian flu in terms of seriousness?

① Much higher ② A little higher
③ Same ④ A little lower
⑤ Much lower

BF5c. How does H7N9 avian flu compare with SARS in terms of seriousness?

① Much higher ② A little higher
③ Same ④ A little lower
⑤ Much lower

BF6. If you were to develop flu-like symptoms tomorrow, where would you seek medical consultation? (can choose 1 item or more)

① Public clinics/A&E department consulting Western medical doctors
② Public clinics/A&E department consulting Traditional Chinese medicine
③ Self-treatment
④ Telephone to a medical professional, such as 12320 hot-line
⑤ Internet
⑥ Others (please specify): _________

⑦ 肯定会

BF2a. 与家庭外的同龄人相比，您认为自己在接下来的 1 个月患上 H7N9 禽流感的机率会是？

① 肯定不会 ② 机率小很多
③ 机率较小 ④ 机率一样
⑤ 机率较大 ⑥ 机率大很多
⑦ 肯定会

BF3. 您认为 H7N9 禽流感是通过哪些途径传播的，请分别作答：

		是	否	不知道
a.	飞沫	①	②	③
b.	空气	①	②	③
c.	手部直接接触（例如：握手）	①	②	③
d.	手部间接接触（例如：门把手）	①	②	③
e.	口-粪便传播	①	②	③
i.	天气冷	①	②	③
k.	远距离空气传播（例如：楼宇间）	①	②	③
l.	与 H7N9 禽流感病人有身体接触	①	②	③
m.	接触了被 H7N9 禽流感病毒污染的物体	①	②	③
n.	密切接触了活禽市场的鸡鸭	①	②	③

BF4. 如果这两天您突然出现了流感症状（例如：发烧、咳嗽、流涕、咽痛），您将？

① 完全不担心 ② 比平常担心少很多
③ 比平常担心少 ④ 与平常一样
⑤ 比平常担心多 ⑥ 比平常担心多很多
⑦ 极度担心

BF4a. 过去 1 周内，您是否曾为患上 H7N9 禽流感担心过？

① 没有，从未考虑过 ② 考虑过，但不担心
③ 有些担心 ④ 很担心
⑤ 一直都在担心

BF5a. 与普通流感相比，您认为 H7N9 禽流感严重程度是？

① 严重很多 ② 较严重
③ 差不多 ④ 较轻微
⑤ 轻微很多 ⑥ 不知道

BF5b. 与 H5N1 禽流感相比，您认为 H7N9 禽流感严重程度是？

① 严重很多 ② 较严重
③ 差不多 ④ 较轻微
⑤ 轻微很多 ⑥ 不知道

BF7. How would you evaluate the current performance of the national government in controlling H7N9 avian flu?

Effectiveness of prevention measures: ______ (0=extremely poor, 5=moderate, 10=excellent)

BF7a. How would you evaluate the current performance of the provincial/city government in controlling H7N9 avian flu?

Effectiveness of prevention measures: ______ (0=extremely poor, 5=moderate, 10=excellent)

Part 6 Preventive Measures

PM1. Did you receive flu vaccine in the past 3 years?

①Yes ②No ③ Don't know

The following questions refer to the past 3 days:

PM2. Did you cover your mouth when you sneeze or cough?

① Always ② Usually
③ Sometimes ④ Never
⑤ Don't know

PM3. Did you wash your hands after sneezing, coughing or touching nose?

① Always ② Usually
③ Sometimes ④ Never
⑤ Don't know

PM3a. Did you wash your hands after returning home?

① Always ② Usually
③ Sometimes ④ Never
⑤ Don't know

PM4. Did you use liquid soap when washing your hands?

① Always ② Usually
③ Sometimes ④ Never
⑤ Don't know

PM5. Did you wear face mask?

① Always ② Usually
③ Sometimes ④ Never (go to PM6)
⑤ Don't know (go to PM6)

PM5d. The reason(s) of your wearing mask: *(can choose 1 item or more)

BF5c. 与非典（SARS）相比，您认为 H7N9 禽流感严重程度是？

① 严重很多 ② 较严重
③ 差不多 ④ 较轻微
⑤ 轻微很多 ⑥ 不知道

BF6. 如果这两天您突然出现了流感症状（例如：发烧、咳嗽、流涕、咽痛），您可能会（可多选）？

① 医院看西医
② 医院看中医
③ 到私人小诊所就医
④ 自我医疗，如服中药、理疗等
⑤ 打专业热线电话咨询， 例如，12320 热线
⑥ 上网查询有关信息
⑦ 其他（请注明）：_________

BF7. 对国家目前防控 H7N9 禽流感的成效，用 0-10 分进行评价，您会给出_____分（0 代表极差，5 代表中等，10 代表非常棒）

BF7a. 对本地目前防控 H7N9 禽流感的成效，用 0-10 分进行评价，您会给出_____分（0 代表极差，5 代表中等，10 代表非常棒）

下面还有几个问题，我们的访问就会结束。

第六部分 预防措施

PM1. 过去 3 年中您曾接种过流感疫苗吗?

①有 ②没有 ③不记得

请注意，以下问题是针对过去 3 天：

PM2. 过去 3 天内，当您打喷嚏或咳嗽的时候是否有遮掩的动作?

① 总是 ② 通常
③ 有时 ④ 从不
⑤ 不记得了 ⑥ 不适用（无打喷嚏或咳嗽）

PM3. 过去 3 天内，当您擦鼻涕、咳嗽和打喷嚏后是否洗手？

① 总是 ② 通常
③ 有时 ④ 从不
⑤ 不记得了 ⑥ 不适用（无擦鼻涕、咳嗽或打喷嚏）

PM3a. 过去 3 天内，您外出回家后是否马上洗手？

① Protect myself ② Protect others
③ Feel secure ④ Other people are wearing masks
⑤ Others: __________

PM6. Did you use serving utensils when dining with others?
① Always ② Usually
③ Sometimes ④ Never
⑤ Don't know

PM7. In the past 7 days did you：(please answer directly with "yes" or "no"), Note: If answered "yes", interviewer should immediately inquiry whether it is because of H7N9 flu.

		Yes, due to H7N9 flu	Yes, but not due to H7N9 flu	No	Don't know
a.	avoid eating out?	①	②	③	④
b.	avoid using public transport?	①	②	③	④
c.	avoid going to crowded places?	①	②	③	④
d.	keep good indoor ventilation?	①	②	③	④
e.	reschedule travel plan?	①	②	③	④
f.	clean or disinfect house more often?	①	②	③	④

PM8. If free H7N9 flu vaccine is available in the coming month, would you consider receiving it?
①Yes ②No ③ Not sure ④ Don't know

Part7 Demographics

SG6. What is your marital status?
① Single ② Married
③ Divorced /separated ④ Widowed
⑤ Refuse to answer

SG7. How many people live in your household, including yourself and domestic helper(s)? ___

SG8. What is your education level?
① primary school and illiteracy ② Middle school
③ High school ④ College and above

SG9. Do you or your family members have the hobbies to raise chicken, duck or homing pigeon in your home?
① Yes ② No

① 总是 ② 通常
③ 有时 ④ 从不
⑤ 不记得了 ⑥ 不适用（无外出）

PM4. 过去 3 天内，您洗手时是否使用肥皂或洗手液？
① 总是 ② 通常
③ 有时 ④ 从不
⑤ 不记得了

PM5. 过去 3 天内，您戴过口罩吗?
① 总是 ② 通常
③ 有时 ④ 从不（跳到 PM6）
⑤ 不记得了（跳到 PM6）

PM5d. 您戴口罩的原因是（可多选）？
① 保护自己 ② 保护他人
③ 感觉安全 ④ 其他人戴，所以我也戴
⑤ 其他原因（请注明）： __________

PM6. 过去 7 天内，您是否出现以下情况？请分别作答：请用"是"或"不是"进行回答。注意，(如果选择"是"，访问员要追问"是""否"因为 H7N9 禽流感)

情况说明		是	否	如"是"，是否因为 H7N9 禽流感	
				"是"	"否"
a.	避免外出用餐?	☐	☐	☐	☐
b.	避免乘坐公共交通工具?	☐	☐	☐	☐
c.	避免到拥挤的公共场所?	☐	☐	☐	☐
d.	保持室内空气流通?	☐	☐	☐	☐
e.	调整了出行计划?	☐	☐	☐	☐
f.	更频繁打扫或消毒房间?	☐	☐	☐	☐

PM7.如果政府下个月为市民免费接种 H7N9 禽流感疫苗，您是否考虑接种？
① 是 ② 否 ③不一定 ④不知道

下面我将再了解您的一点个人情况，我们的访问就结束了。

SG10. Did you travel to other province (or prefecture) in the past month?

a) ① Yes ② No

b) If "Yes", please specify the destination of your last travel: ___________________

c) When did you travel back: ___(month) ___(day) (or ___days before)

SG11. Did you travel abroad in the past month?

a) ① Yes ② No

b) If "Yes", please specify the country of your last travel: ___________________

c) When did you travel back: ___(month) ___(day) (or ___days before)

SG12. What is your household total income from employment?

① Less than 2,000	② 2,001—4,000
③ 4,001—6,000	④ 6,001—8,000
⑤ 8,001—10,000	⑥ 10,001—15,000
⑦ 15,001—20,000	⑧ 20,001—25,000
⑨ 25,001—30,000	⑩ 30,001—40,000
⑪ 40,001or more	⑫ No income

SG13. Name (Chinese) : ________________________ *(If refuse, please record the salutation)

SG14. Would it be possible to contact you again in a few weeks for a follow-up survey?

① Yes ② No ③ It depends

第七部分人口资料

SG6. 您的婚姻状态？

① 单身	② 已婚
③ 离异/分居	④ 丧偶
⑤ 拒绝回答	

SG7.家里/住处有多少人共同居住，包括保姆等： ___人

SG8. 您的教育程度？

① 从未接受过学校教育	② 小学
③ 初中	④ 高中
⑤ 大学及以上	

SG9.您或您的家人是否有在家饲养鸡、鸭或鸽子？**（仅问 6 个城市的居民）**

①是 ②否

SG10. 上个月您是否离开本地去过别的地市/省份？（根据访问的不同城市分别设置题面）

a）①是 ②否

b）如果选"是"，最近一次的目的地:____________________（国内问到地市，国外问到国家）

c）您何时返回：___月___日 （或者 ___天以前）

SG12. 您的家庭月均收入是多少人民币？（不与家人同住者，仅问个人的月均收入）

① 不足 1,000	② 1,001—2,000
③ 2,001—3,000	④ 3,001—4,000
⑤ 4,001—6,000	⑥ 6,001—8,000
⑦ 8,001—10,000	⑧ 10,001—15,000
⑨ 15,001—20,000	⑩ 20,001—30,000
⑪ 30,001 以上	⑫ 无收入
13 不清楚	14 拒答

SG14.可以在未来几个星期内再次随访您吗？

① 可以 ②不可以 ③看情况，在我方便时

SG13. 请问您贵姓（中文）: __________先生/女士
